# Characterization of the Interface Between Coating and Fibrous Layers of Paper

**DOI:** 10.1007/s11242-018-1183-2

**Published:** 2018-10-30

**Authors:** H. Aslannejad, S. M. Hassanizadeh, M. A. Celia

**Affiliations:** 1 Environmental Hydrogeology Group, Department of Earth Sciences, Universiteit Utrecht, Princetonlaan 8a, 3584 CC Utrecht, The Netherlands; 2 Department of Civil and Environmental Engineering, Princeton University, Princeton, NJ 08544 USA

**Keywords:** Coated paper, Interface, Transition zone, Pore-scale modeling, FIB-SEM, Thin layer, Characterization

## Abstract

Coated paper is an example of a multi-layer porous medium, involving a coating layer along the two surfaces of the paper and a fibrous layer in the interior of the paper. The interface between these two media needs to be characterized in order to develop relevant modeling tools. After careful cutting of the paper, a cross section was imaged using focused ion beam scanning electron microscopy. The resulting image was analyzed to characterize the coating layer and its transition to the fibrous layer. Such image analysis showed that the coating layer thickness is highly variable, with a significant fraction of it being thinner than a minimum thickness required to keep ink from invading into the fibrous layer. The overall structure of the coating and fibrous layers observed in this analysis provide insights into how the system should be modeled, with the resulting conclusion pointing to a specific kind of multi-scale modeling approach.

## Introduction

Cellulose-based papers are the main substrate for the printing industry as well as the main component of a new generation of “biodegradable” medical diagnostic devices. Similar considerations apply to microfluidic kits for diagnostic devices (cf. López-Marzo and Merkoçi 2016), where movement of water into and between cellulose fibers is largely controlled by the hydrophilic property of the fibers. In inkjet printers, a cartridge delivers tiny droplets of ink (Pico-liter in size) on the paper surface. As soon as a pico-liter size droplet of ink reaches the paper, it starts penetrating into the porous substrate.

Uncoated paper is an anisotropic porous medium, which consists of bundles of fibers crossing over each other in a planar orientation. The fibrous medium is commonly impregnated by granular mineral materials as filler. Observations have shown that penetrating liquid in a fibrous layer first follows the direction of fibers and wets them. Then, the pore space between fibers is filled up with the liquid (Aslannejad and Hassanizadeh 2017).

In order to reduce the ink penetration into the fibrous layer and produce a high print quality, coated papers are often used. The added coating material is normally an isotropic granular medium, which has pores in the range of only a few hundred nano-meters. The small pores produce suitable conditions for sucking in the droplet from the surface of paper in a relatively uniform penetration and spreading pattern. A uniform final pattern is desirable from the point of view of print quality.

Paper surface roughness and application of the coating layer on different basesheets have been studied previously. Gane et al. (1991) used optical imaging techniques to study the effect of fiber furnish on the coating structure, roughness and coverage of the paper. They reported that the aqueous coating color caused a relaxation in surface profile of the thermomechanical pulp basesheet and yielded an uneven coating distribution and rough uncalendered coated paper. The ground wood basesheet retained stability in its surface profile during the coating process, although the basesheet itself is a rough basesheet. In case of using pressurized ground wood basesheet, a smooth coated sheet resulted with a relatively uniform distribution of the coating layer.

In another work, Gane and Hooper (1989) used coating thickness analysis and frequency transform procedures to study basesheet surface profile change during paper coating application. They showed that the relaxation of the basesheet depends on the type of coating pigments, their size distribution, their rheology and dewatering interaction between the coating and basesheet. They also concluded that dewatering characteristics were determined by base absorbency, pigment particle packing, and suspension of the fluid viscosity.

The print quality is partly determined by the spatial variation of paper properties and ink density. If they have irregularities, it leads to non-uniform ink absorbency across the surface of the paper, which is referred to as mottle in printing. Gane (1989) used Walsh transform spectrum to identify the main reason for mottle in a printed sample. He established that the binder migration (i.e., the redistribution of binder by penetrating ink) is the main cause of mottle. He showed that binder migration depends on coating distribution and variations in the basesheet absorbency.

Lamminmäki et al. (2011a, b) tried to clarify the effect of ionic charge distribution in the coating layer on dye fixation properties. They chose surface inert organosilica and modified calcium carbonate as model coating structure. Non-ionic polyvinyl alcohol (PVOH) and anionic polymer were added as binder. Then, the surface was treated by applying a cationic polymer. The ab/adsorption of the colorant part of ink was evaluated using UV–VIS spectroscopy. They showed that addition of PVOH and anionically dispersed coating increased colorant fixation. In addition, cationic additive application slowed down the ink imbibition into paper. Altogether, this resulted in less bleeding and improved water fastness properties.

In high speed inkjet printing process, the pore network of the coating layer plays an important role in ink uptake. Lamminmäki et al. (2011a, 2012) studied the possibility of lowering the thickness of the coating layer and reducing bleeding, which is when the ink spreads during setting. They showed that at the early stage of ink arrival on the surface of coated paper, capillary flow is dominant. Nevertheless, just four milliseconds after application of ink, permeability plays a more important role. Moreover, the pigment type and binder amount in the coating layer were found to have no influence on results.

In the case of coated paper, we should characterize not only the two different thin porous layers—the coating layer and the fibrous layer—but also the interface between them. In the case of the fibrous layer, some studies were done using X-ray microtomography imaging techniques to extract the paper’s three-dimensional (3D) pore space (du Roscoat et al. 2005, 2008). The extracted domain has been used in direct simulations using Lattice Boltzmann methods (Hyväluoma et al. 2007; Ramaswamy et al. 2004; Rosenholm 2015; Järnström et al. 2010). In some simpler approaches, the fibrous layer has been considered as an array of similar structure units representing the real pore space (Salmas et al. 2001; Washburn 1921; Schoelkopf et al. 2002).

Ghassemzadeh and Sahimi (2004a) reported a method to determine the size distribution and connectivity of fibrous layers of paper using two-dimensional (2D) cross-sectional SEM images. They used the results to conduct pore-network simulations of fluid flow into paper during coating (Ghassemzadeh and Sahimi 2004a).

In another work, Ghassemzadeh and Sahimi (2004b) developed a statistical approach to characterize paper structure using the distribution of radius and length of pores between fibers. Based on extracted data, the paper layer was represented by a 3D network of interconnected channels. Then, they used the network to determine the effective permeability tensor of paper.

Ridgway et al. (2002) and Kettle et al. (2010) studied the effect of pore-network structure on dynamic imbibition into paper. They used the Bosanquet equation in a 3D network simulator (called Pore-Cor). In their work, film flow along the fibers was not considered. They concluded that over a short time interval, smaller pores were filled faster than larger pores, which is not in agreement with Washburn equation. In addition, they found that the aspect ratio of a pore, defined as the ratio of length to radius, plays an important role in the filling rate of the pore.

**Fig. 1:**
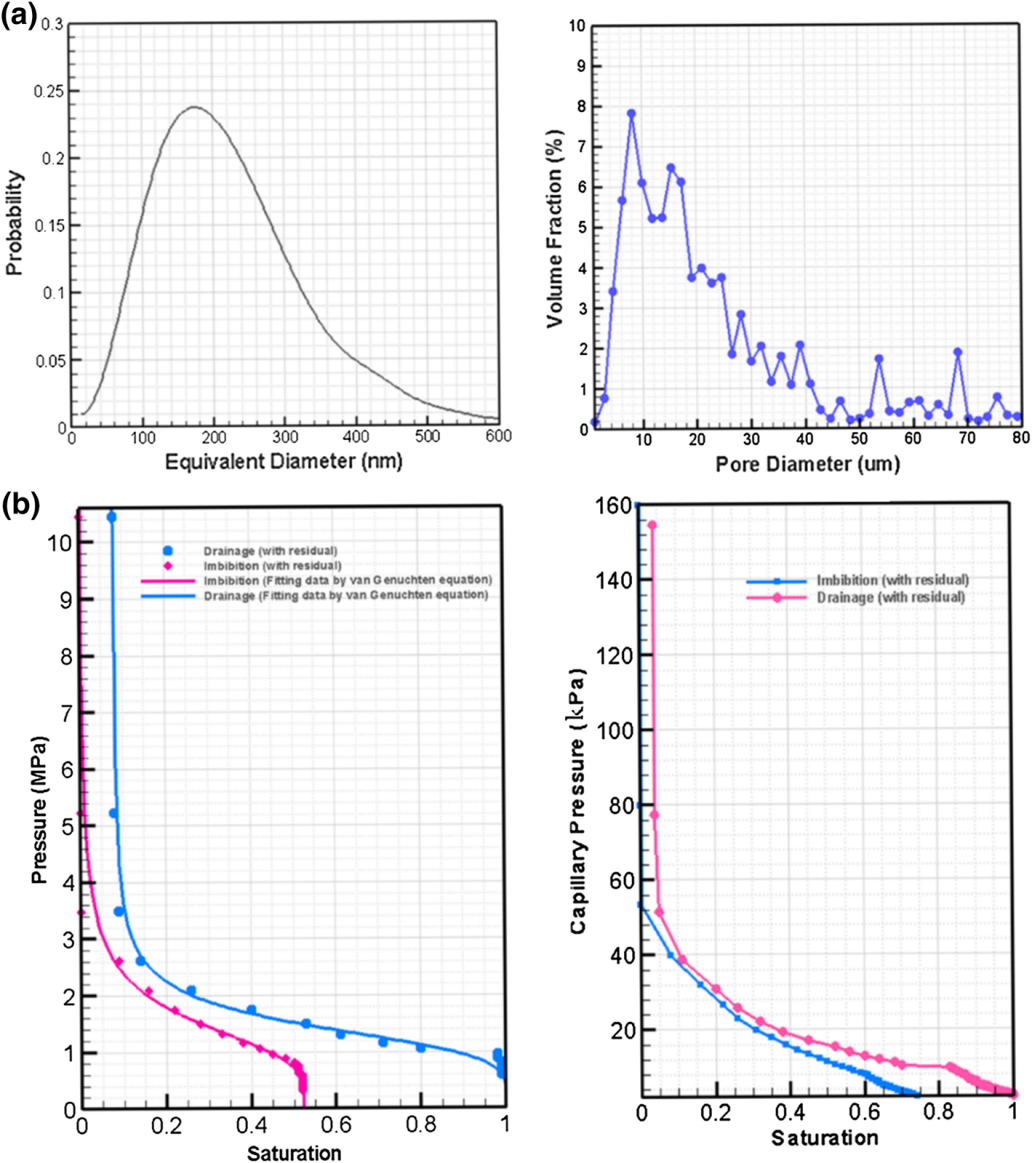
**a** Pore size distribution of the coating and fibrous layers, and **b** capillary–pressure–saturation curves of the coating and fibrous layers (Aslannejad et al. 2017; Aslannejad and Hassanizadeh 2017)

In an earlier work, using X-ray microtomography, the 3D structure of a fibrous layer was extracted and reconstructed (Aslannejad and Hassanizadeh 2017). Then, a pore-morphology method was used to obtain the pore size distribution, and curves of capillary pressure and relative permeability, as a functions of fluid saturation. Recently, focused ion beam scanning electron microscopy (FIB-SEM) imaging techniques were used to acquire and reconstruct the 3D pore network of the coating layer (Aslannejad et al. 2017). The extracted network was used for pore-network modeling and determination of hydraulic properties of the coating layer. Pore size distribution of the coating and fibrous layers were determined. Using the pore-morphology method, capillary pressure–saturation curves of the two layers were also determined. Graphs of pore size distribution and capillary pressure–saturation curves are shown in Fig. 1. As expected, capillary pressure–saturation curves show much higher capillary pressure values for the coating layer. This is related to much smaller mean pore size of the coating layer (Fig. 1a). To the best of our knowledge, an imaging of the transition from coating layer to the fibrous layer in a coated paper has not been done up to now. Therefore, here we are focusing on not the coating and fibrous layer but on the transition area from the coating to the fibrous layer.

In the case of multi-layer porous media, like the coating layer–fibrous layer system, there is always a contact interface between the two layers. Depending on the details of the spatial structure of this interface, it might be modeled as a uniform planar surface of discontinuity in material properties, a zero-thickness interface with its own properties, or as a finite-thickness transition zone between the layers.

In this paper, we focus on understanding the characteristics of the interface between the coating layer and the fibrous layer in a coated paper system and its effect on ink absorption into the coated paper. We first present a brief overview of the pore space and properties of coating and fibrous layers. Then we provide information about the interface between the two layers. We have obtained this information with the aid of imaging techniques and image analysis. This includes measurement of the coating layer thickness as a function of location along the interface. From that analysis, we generated a coating thickness histogram for a relatively large cross section of the coated paper. Based on this information, we discuss whether the coating layer and the interface with basesheet can be simulated by traditional macro-scale modeling approaches. We also provide suggestions for including the effect of variation of the coating layer thickness in computational models. Finally, we have estimated the minimum thickness of coating layer required in order to ensure that water does not reach the fibrous layer. Identifying this minimum layer thickness is a novel contribution to the understanding of the role of coating layer in print quality.

## Materials and Methods

### Paper Samples

**Fig. 2:**
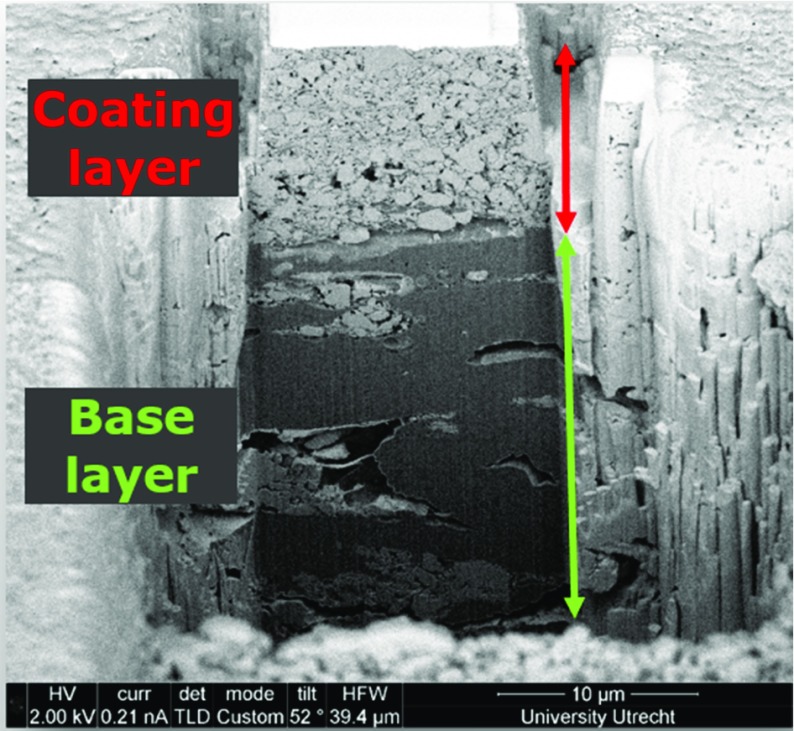
Cross-sectional view of the coated paper showing the coating layers and part of the fibrous layer

In this work, we studied samples of a coated paper (Magno glass, Sappi, Germany), which is primarily an offset printing grade and is not usually used in inkjet printing. However, as we are interested in studying the coating-basepaper interface and liquid transfer through it, we have used it to illustrate our characterization method and analysis approach. Our results and approach can be used to study papers that are optimized for inkjet printing. The paper cross-sectional view is shown in Fig. 2. As seen in the figure, the coated paper has a base layer made of cellulose fibers covered (on both sides) with coating layer consisting of a pre-coat and a topcoat, these are clearly delineated by particle size. In addition, the space between the pigment particles in the pre-coat is largely filled by soluble binder, whereas the topcoat contains particulate binder. This is typical of double coated gloss offset papers. The coating layer is mainly (88% mass fraction) made of compressed CaCO3 powder with an average thickness of 15 µm, porosity of 34%, and mean pore size of 180 nm. The amount of binder present in the layer is about 8% by mass, or 20% by volume. This is a significant volume and will affect the connectivity of the coating layer pore structure (Aslannejad et al. 2017).

In order to compare the coated and uncoated papers, samples of an uncoated printing paper, Ziegler Z-Plot 650 (Ziegler papier AG, Germany), were analyzed. The paper consisted of a single layer with an average thickness of 150 µm, made of filler-free cellulose-based fibers. This paper has a porosity of 50% and mean pore size of 12 µm (Aslannejad and Hassanizadeh 2017). It should be mentioned that in fibrous layers, normally there are two directions, machine and perpendicular directions. Most of the fibers lie in the plane of these two sheet directions, with the majority aligned in the machine direction. This is also the case in the sampled analyzed. Table 1 shows detailed information about the samples used in this study.

**Table 1:** Properties of coating layer, coated paper, and uncoated paper

	Thickness (µm)	Porosity	Mean pore size	Permeability* (mDarcy)	Grammage (gr/m2)	References
Coating layer	15	34%	180 nm	0.1	–	**
Coated paper	85	–	–	–	115	***
Uncoated paper	150	50%	12 µm	5500	90	****

*In thickness direction

**Aslannejad et al. (2017)

***https://www.sappi.com/magno

****Aslannejad and Hassanizadeh (2017)

### Imaging

The imaging of coated paper was done using a Nova Nanolab 600 focused ion beam scanning electron microscope (FIB-SEM) (FEI Company, Eindhoven, Netherlands). Typical imaging conditions were 2 kV and 0.21 nA. The FIB acceleration voltage was 30 kV for all processes (e.g., deposition, rough cutting, polishing); the current density was varied according to the required process. For more details, readers are referred to Aslannejad et al. (2017).

**Fig. 3:**
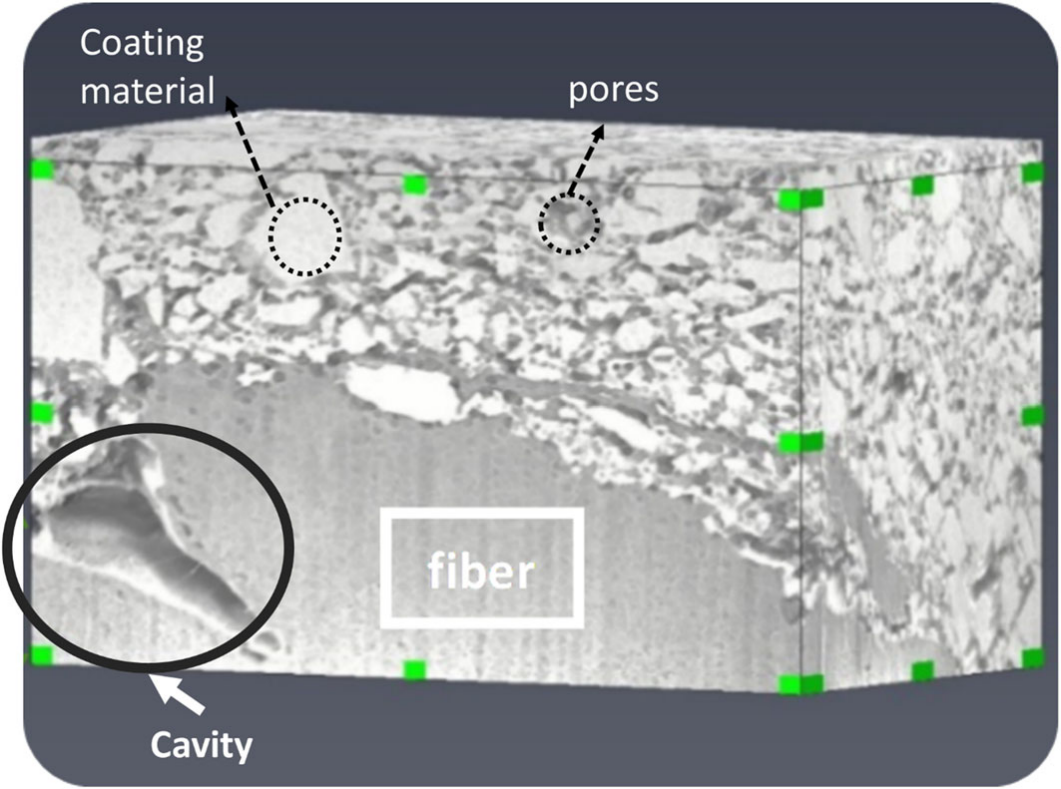
3D domain of coated paper showing coating layer as well as the connection to the fibrous layer (Observation domain has dimensions of 20 × 15 × 15 µm)

Note that the FIB-SEM could not be used for imaging the full thickness of fibrous layer. The maximum practical domain size to be imaged by FIB-SEM is a cube of 20 × 15 × 15 µm^3^ (the cube in Fig. 3). As shown in Fig. 3, the imaged domain contained not only coating material but also some fibers of the fibrous layer.

### Image Analysis

From the acquired images and using Avizo software (Fire edition, FEI, Oregon, US), we reconstructed the paper pore space. First, a median filter was applied to remove the imaging noise. Then, alignment and thresholding modules with appropriate adjustments were applied to extract binary three-dimensional (3D) structure of the solid phase. In the extracted images shown in Fig. 3, coating material, fibers and pores can be distinguished.

### Determination of Coating Layer Thickness Along Paper Cross Section

**Fig. 4:**
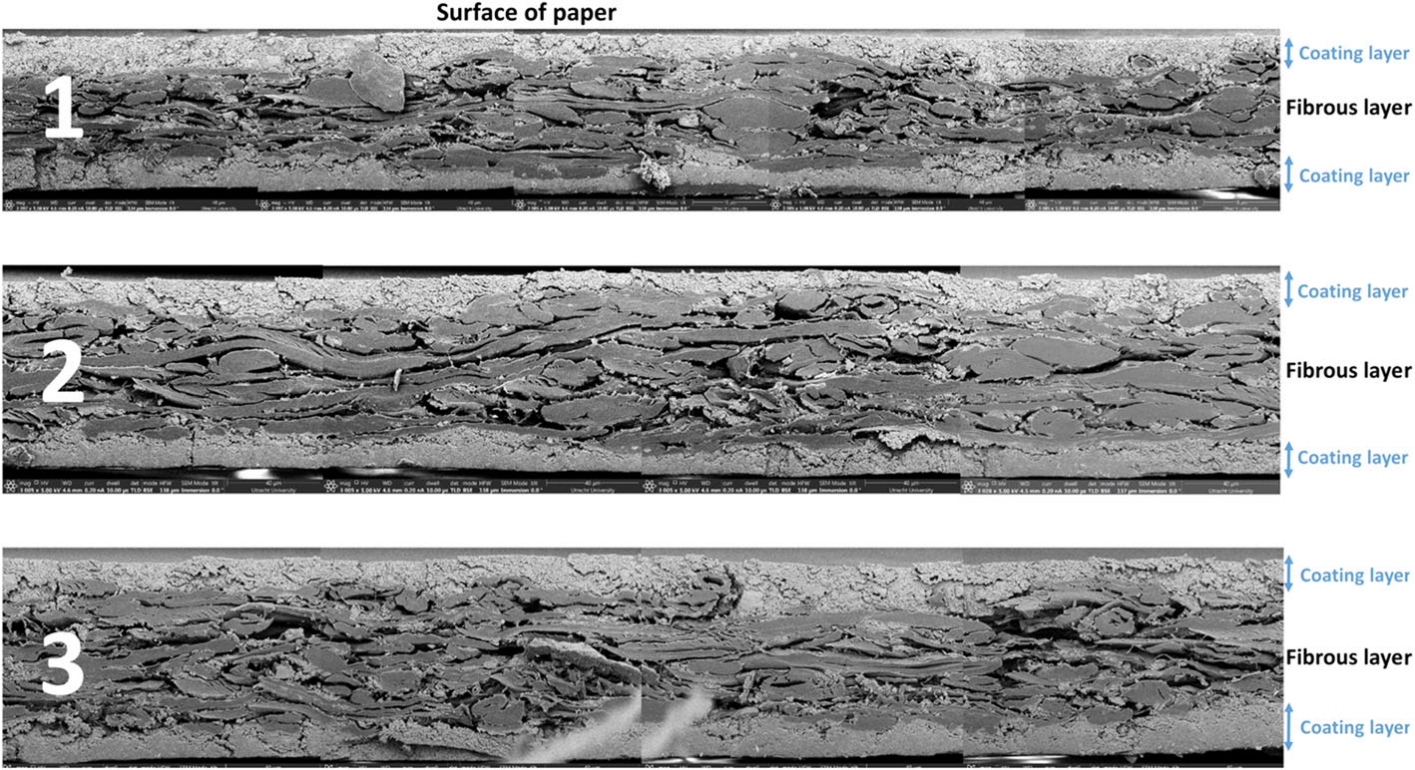
Cross section of a piece of coated paper 4 mm long, obtained by SEM imaging

In order to study thickness of the coating layer along the coated paper cross section, the paper sample was cut using a microtome (Clamp-able Manual Microtome MT.5503). This resulted in a sharp cross section of sample without mixing layers of paper or damaging any fiber. Then, the cut edge was imaged using FIB-SEM. In total, 20 cross-sectional locations were imaged, each about 200 µm long. Then, the images were put together to obtain a relatively long cross-sectional view of coated paper, about 4 mm long. Figure 4 shows the resulting cross-sectional view of the coated paper.

### Print Quality

To study the role of the coating layer and its required minimum thickness for keeping the ink inside it and preventing it from reaching the fibrous layer, two different printing qualities were considered: 600 and 1200 Dots per Inch (DPI). In inkjet printing, each printed character on the paper is made of several ink droplets. The spacing of droplets forming a character is usually given as DPI. In the case of 600 DPI, droplets of ink with diameter of about 10 µm are jetted onto the paper with center-to-center distance of about 18 µm (Lamminmaki et al. 2009).

**Fig. 5:**
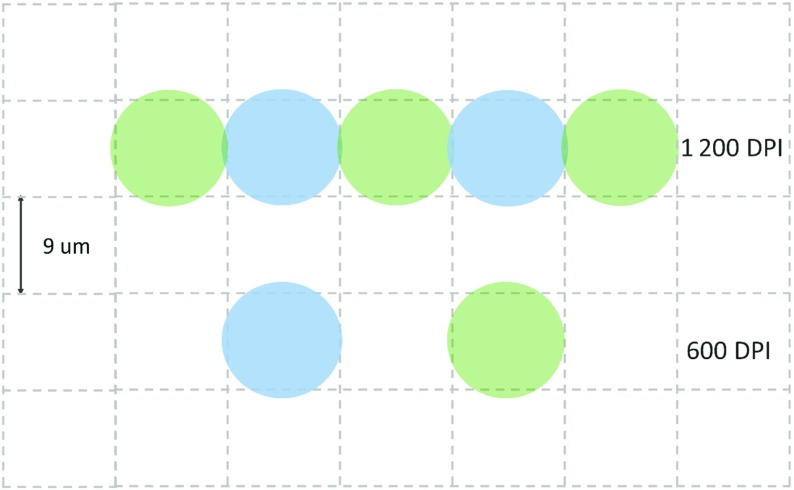
Schematic representation of droplet spacing jetted on paper for the cases of 600 and 1200 DPI print qualities

In the case of 1200 DPI, droplets are usually jetted closer to each other, which results in higher print resolution. For instance, droplets of ink with diameter of 10 µm are jetted with spacing of 9 µm. Figure 5 shows a schematic representation of 600 and 1200 DPI print qualities. As seen in the figure, the droplets usually have small overlap in the case of 1200 DPI.

**Fig. 6:**
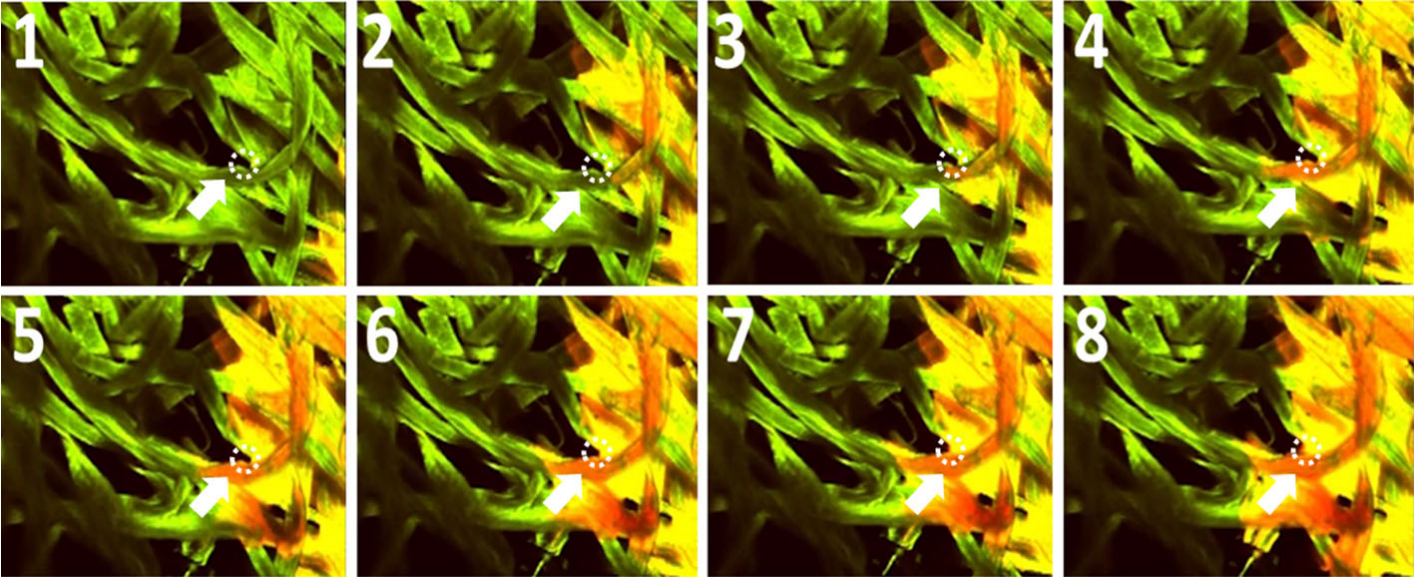
Snapshots of water movement injected into a fibrous layer. Images of penetration are obtained using a confocal laser microscope (Nikon A1^+^) (Aslannejad and Hassanizadeh 2017). Numbers indicate time steps; green and orange colors represent dry and wet fibers, respectively; yellow color shows intermediate saturation. The arrows are pointing to an area of high saturation ahead of the main water front; the water moves on and into fibers and then fills the pore space between fibers

In order to understand the consequences of water-based ink reaching the base layer (fibrous layer), water movement into the fibrous base layer needs to be studied (as, e.g., in Aslannejad and Hassanizadeh 2017). Fibers are highly hydrophilic and as soon as water reaches any of them, water starts to creep on their surfaces and penetrates them. This is shown in Fig. 6, where snapshots of images of penetration of water injected into a fibrous layer are shown. Water was introduced from the right side and images were obtained using a confocal laser microscope (Nikon A1^+^). We see that saturation has gone up in fibers (dark orange color) ahead of the main front (yellow color).

For a better print quality, all fibers should be sufficiently covered with the coating layer; otherwise ink may come into contact with fibers and get transported into the fibrous layer. The main detrimental effect of ink penetrating the fibrous layer is that the film flow along the fiber surface leads to wicking and a spider-leg like effect on the print. The swelling of fibers is another important effect, which may change the thickness of paper; this could potentially cause tray blockage during the printing process. Since the fibrous layer is made of elongated fibers, its surface commonly has a relatively significant roughness. The roughness has a major effect on the thickness and quality of the coating layer. As seen in Fig. 4, tiny particles of the coating material cover the fibrous layer and form a relatively smooth coating layer. Rougher fibrous layers need larger coating layer thickness to cover all fibers.

Although the permeability value of the coating layer is low (Aslannejad et al. 2017) and the droplet stays for a while on the paper surface and evaporation plays a role, the coating layer should have enough thickness to keep all the remaining ink liquid within the layer. The required minimum thickness of coating layer for absorbing the liquid part of ink depends on the volume of ink in the droplet: a larger volume needs more thickness to handle the liquid part. For example, if we ignore evaporation, in the case of 600 DPI printing, where an ink droplet has a diameter of 10 µm, the coating layer should have a thickness of 18 µm. In making this rough estimate, we have assumed that the droplet has a spherical shape and invades the coating layer (with porosity of 34%) cylindrically. The assumption that the liquid penetrates cylindrically was made only in order to make a rough estimate. We know from previous works that the liquid absorbs via a preferred pathway and many of the coating layer pores remain unfilled prior to the liquid reaching the base paper [see, e.g., Ridgway and Gane (2002), Schoelkopf et al. (2002) and Aslannejad et al. (2018)].

## Results and Discussion

In this section, the results of the image analysis are presented. This includes the spatial distribution of the coating layer thickness, its associated spatial correlation structure, and the frequency of occurrence of coating thicknesses below the estimated minimum required thickness. We refer to locations with thickness below the minimum amount as “weak points.” In addition to the spatial analysis, we also discuss an approach for modeling all layers of the coated paper.

### Distribution of Paper Coating Layer Thickness and Weak Points

**Fig. 7:**
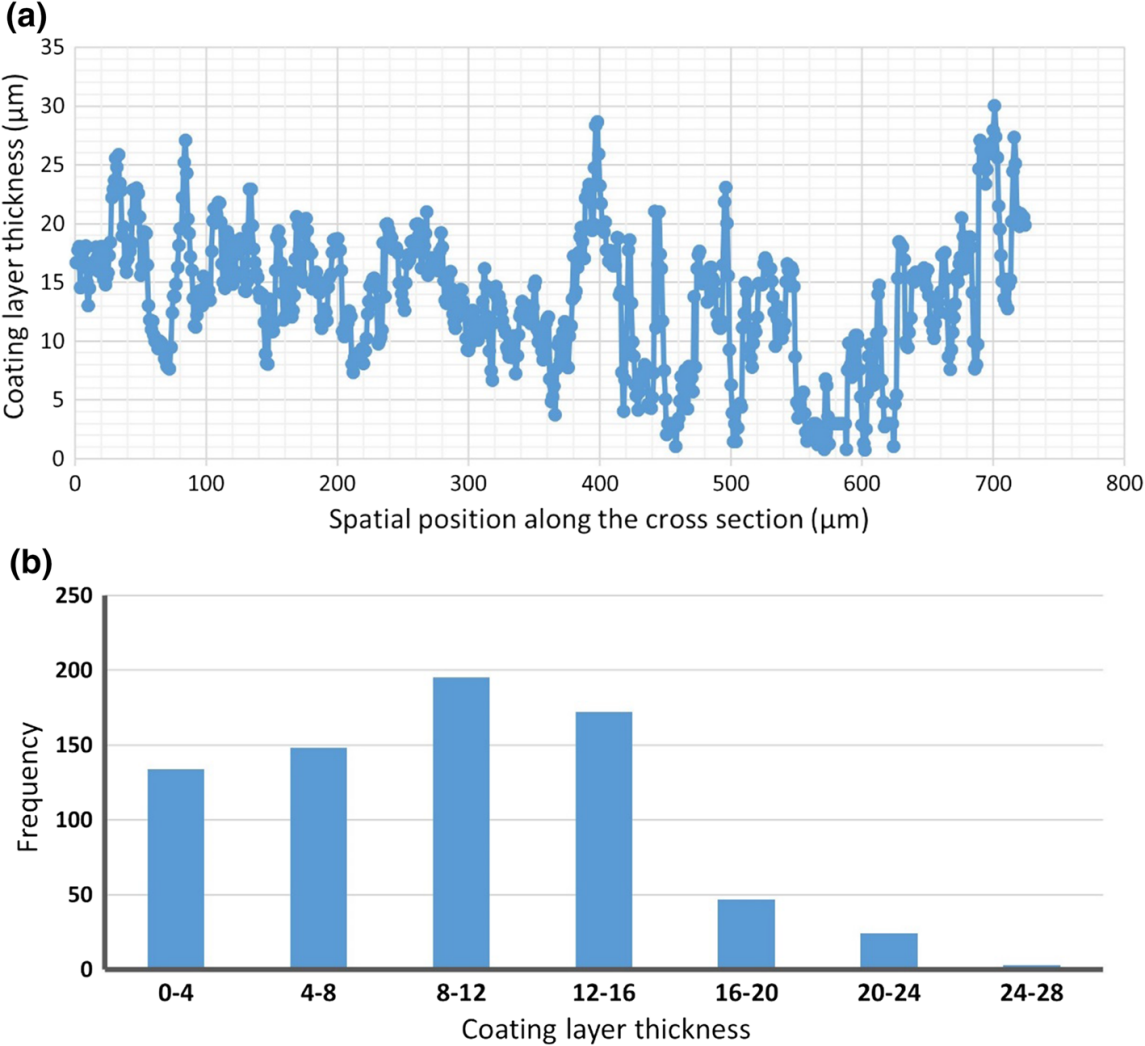
**a** The coating layer thickness as a function of (one-dimensional) spatial location and **b** coating layer thickness histogram

The cross-sectional images were analyzed to identify the thickness of the coating layer along the entire length of the cross section. This provides thickness as a function of location. That function is plotted in Fig. 7a, with a summary histogram of the data plotted in Fig. 7b. These data show a maximum thickness of 30 µm and a minimum thickness of one micron, with a distribution weighted toward the lower thickness values. The data show that most of the thickness values fall below the estimated target value of 18 µm. This indicates that the paper fails to satisfy the requirements of coating thickness to produce high quality printing results for a 10-µm-diameter droplet. However, we must note that this failure was expected for this the paper type studied in this work, as it was designed for offset printing and not inkjet printing technology.

**Fig. 8:**
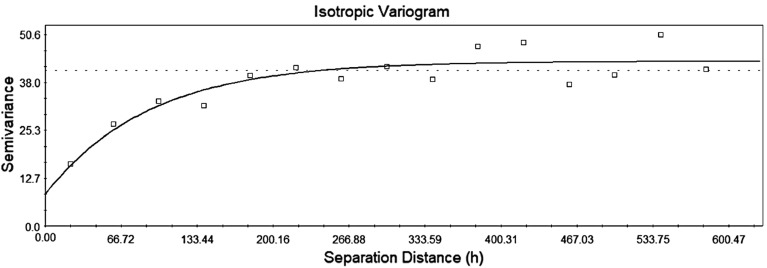
Semivariance diagram showing correlation length

In order to estimate the spatial correlation structure of the thickness function, a semi-variogram was calculated, with the results shown in Fig. 8. The variance is 40 µm while the correlation length is around 200 µm. This shows that the thickness has significant spatial variability and there is some level of spatial structure in the variability of the thickness function. The correlation length of 200 µm could well relate to the floc site of fibers due to the sheet formation. The correlation length, as described by Gane et al. (1996), is a primary property of a basesheet when designing a suitable coating strategy for both coverage and print uniformity. In addition to that, all of these might be useful parameters in building models to analyze fluids flow in such complex structures.

### Modeling of Ink Imbibition into Coated Paper

There are two main modeling approaches for modeling ink imbibition into a layer of coating material. One is the traditional three-dimensional macro-scale (or continuum) model of unsaturated flow. The other one is pore-scale modeling, including direct simulation or pore-network modeling methods.

For macro-scale models to be applicable, it is commonly stated that one should be able to identify a Representative Elementary Volume (REV), over which average quantities can be defined. An REV is required to contain a large number of pores in each direction. In addition, its size must be much smaller than dimensions of the domain that is to be modeled. In the case of (coated) paper, the two criteria are mutually excluding; if the REV is to be much larger than the pore size, it cannot be much smaller than the layer thickness. This is even more so in the case of coated paper studied here.

As explained in Sect. 2.1, the coating layer consists of a pre-coat and a topcoat, whose average thicknesses are 10 and 2 µm, respectively. Thus, even though the mean particle sizes is 100 nm, it is not feasible to consider the coating layer as a three-dimensional continuum, as the basic criteria for the definition of average macroscopic quantities cannot be satisfied.

In addition to that, there are places where the coating thickness is very small. This may happen if the coating penetrates into the basepaper and/or is shunted away from high points, leaving fibers wholly uncoated. Then, as explained earlier, as soon as liquid reaches any fiber, it will be absorbed and the fiber will start to swell. Therefore, in modeling the coated paper, it seems necessary to model individual fibers embedded in the coating layer. This, however, will not be straightforward in the framework of a macro-scale modeling. Alternatively, a full pore-scale description, resolving pores in both the coating layer and the fibrous layer, could be pursued. The swelling behavior of the fibers is important, so special attention needs to be paid to the detailed topology, geometry and structure of the fibers. For example, as fibers have a micro-porosity; this probably leads to a kind of dual-porosity approach. Overall, with this level of detail and the complex spatial structure of the coating layer and the fibers, this is a major modeling challenge and represents an important and interesting area for further research.

## Conclusions

The thickness of the coating layer of a sample of coated paper was analyzed by precisely cutting the sample and then imaging the resulting cross section using FIB-SEM technologies. Subsequent analysis of the images provided a detailed quantification of the spatial structure of the coating layer. The thickness of the coating layer is highly variable, with a significant fraction (80%) showing a thickness below the estimated minimum thickness required to prevent ink from reaching the fibrous layer. Analysis of the variability and spatial structure of the thickness showed a variance of 40 µm and a correlation length of 200 µm. This is a primary property of a coated paper when designing a suitable coating strategy for both coverage and print uniformity.

This kind of analysis provides detailed insights into the effectiveness of the coating layer, and can form the basis of detailed modeling studies for this kind of layered system. Because the coating layer is made of two thin layers, each containing limited pores in the cross-sectional direction, a continuum-scale simulation does not seem appropriate for the coating layer. Similarly, the complex nature of the fibrous layer also fails to satisfy criteria for continuum equations. In both the coating and fibrous layers, enough REVs along the thickness, which is needed for the applicability of continuum-scale modeling, cannot be identified. However, a pore-scale model should be able to include details of the fluid flow through the coating layer of paper and to couple that with the fibrous layer below. The discrete nature of the fibers and their importance in the definition of the overall geometry of the system suggests that these fibers, and the pore spaces between them and within them, need to be modeled discretely. Because swelling of fibers when contacted with the invading wetting fluid is an important consideration, the micro-porous fibers themselves need proper resolution, leading to a multi-scale model. This will be a significant and very interesting modeling challenge.

This approach of detailed imaging with associated image analysis can also be useful for other layered system, where it can also be used to guide in the development of appropriate modeling tools. Based on layer thicknesses, their REV sizes, and their connection (with or without overlap), a proper modeling approach can be identified, based on appropriate measures that come directly from the image analysis.
